# Recombinant expression a novel fibronectin—collage fusion peptide modulating stem cell stemness via integrin β3

**DOI:** 10.1007/s00253-022-11965-4

**Published:** 2022-05-20

**Authors:** Xin Luo, Dezhi Geng, Qirong Zhang, Tao Ye, Yifan Zhang, Ziyi Li, Yadong Huang, Qi Xiang

**Affiliations:** 1 Institute of Biomedicine and Guangdong Provincial Key Laboratory of Bioengineering Medicine, Jinan University, Guangzhou, 510632 China; 2 Biopharmaceutical R&D Center of Jinan University, Guangzhou, China

**Keywords:** Recombinant human fibronectin, Collagen fusion peptide, Integrin β3, Molecular docking, Stemness of stem cell

## Abstract

**Abstract:**

Constructing bionic extracellular matrix (ECM) is an attractive proposition for tissue engineering and clinical regeneration therapy involving the stemness of stem cells. Here, a novel recombinant protein fibronectin-collagen peptide (FCP) was designed to modulate the function of ECM expressed by *Picha. pastoris strain X33*. This FCP promotes cell migration and adhesion and maintains rBMSC stemness by binding integrin β3. Its effects were blocked by both integrin β3 siRNA and the integrin β3 inhibitor Cilengitide. A template-independent ab initio prediction modeling approach is the best approach to construct a stable FCP protein model, which predicts the binding sites between FCP and integrin β3. FCP may be used in the in vitro culture and clinical regeneration of stem cells that highly express integrin β3, such as hematopoietic stem cells. The study provides information on the molecular structure of FCP and its bioactivity, which can be used to design new compounds.

**Key points:**

• *Design a novel recombinant fibronectin-collagen peptide biomimetic ECM.*

• *FCP promotes cell adhesion, migration, and proliferation.*

• *Predicted and verified FCP structure and affinity with integrin β3.*

• *FCP binds integrin β3 to maintain rBMSC stemness.*

**Supplementary Information:**

The online version contains supplementary material available at 10.1007/s00253-022-11965-4.

## Introduction

Stem cells play an important role in tissue engineering and clinical regeneration therapy due to their ability to self-replicate and potential to differentiate into multiple cell lines, called stemness (Keating 2012). The clinical demand for stem cells from various sources far exceeds their supply (Van Zant and Liang 2012). In this regard, an effective solution may be to obtain stem cells through in vitro culture. However, multiple passages of in vitro cultivation gradually decrease the therapeutic properties of stem cells by weakening their proliferation and multipotent differentiation potential, eventually leading to treatment failure (Truong et al. 2019; Zaim et al. 2012). Therefore, developing more substances that can maintain or even enhance the stemness of stem cells during large-scale in vitro culture is important for their clinical applications.

Extracellular matrix (ECM), a noncellular three-dimensional macromolecular network composed of collagens, fibronectin, elastin, and several other glycoproteins (Ishihara et al. 2014; Isomursu et al. 2019), exerts essential functions on the proliferation and differentiation of stem cells (Du et al. 2011). Extensive research and testing have shown that collagen promotes stem cell migration and adhesion (Sorushanova et al. 2019). Fibronectin promotes cell adhesion similar to collagen, and it also fixes collagen to maintain the ECM stability. In addition, the ECM communicates with cells through integrins located on the surface of the cell membrane, such as αvβ3, αvβ1, and αvβ6, via Arg-Gly-Asp (RGD) sequences (Pierschbacher and Ruoslahti 1984). FN10, a part of the fibronectin type III module, contains RGD sequences that specifically bind αvβ1 to guide the fate of the cells (Bharadwaj et al. 2017; Hocking et al. 1996). Moreover, collagen and fibronectin support the growth of human embryonic stem cells (hESC) without the use of a feeder layer under certain conditions (Lu et al. 2006) and play a role in maintaining the stemness of stem cells (Akhir and Teoh 2020; Thaweekitphathanaphakdee et al. 2019). Notably, as the main strain matrix element, collagen relies on fibronectin, fibronectin-bound, and collagen-bound integrins to complete its corresponding functions (Kadler et al. 2008; Kubow et al. 2015). Meanwhile, natural collagen and fibronectin from animal sources have some problems such as unstable quality and difficult dissolution (Davison-Kotler et al. 2019). In contrast, the recombinant proteins based on collagen and fibronectin and prepared by synthetic biology possess the excellent characteristics of high yield, easy amplification, low cost, and not easily contaminated by mammalian pathogens. These characteristics are slowly being recognized as contributors leading to the future application of recombinant proteins based on collagen and fibronectin in regenerative medicine (Ferrer-Miralles and Villaverde 2013). Therefore, it is necessary to use genetic engineering technology to synthesize a novel functional protein that acts as a biomimetic ECM for stem-cell therapy.

Integrin plays an important role in the long-term ex vivo culture of stem cells. As a member of the integrin family, integrin β3 is a transmembrane receptor that mediates interactions between cells and the ECM and which widely exists on the surface of hematopoietic stem cells and bone marrow mesenchymal stem cells (Pei et al. 2011; Umemoto et al. 2012). The integrin-αvβ3 found on hematopoietic stem cells (HSCs) plays an important role in maintaining stem cell activity by regulating stem cell function and affecting stemness through signal transduction (Umemoto et al. 2012; de Graaf and Metcalf 2011; Ishihara et al. 2014). These integrin functions are dependent on specific ECM ligand–receptor interactions and the specific molecular interactions that lead to cytoskeletal changes that result in different migratory behavior or changes in growth and differentiation (Hynes 2002; Isomursu et al. 2019). It is interesting to design innovative proteins based on the functional characteristics of the principal ECM structural proteins that contain specific ECM ligands, such as RGD, and which can bind with integrin β3 to de-anchor and adsorb cells.

Here, we reported a recombinant protein FCP improved according to the early exploration of our laboratory. FCP structure and functions, including binding to integrin β3, were predicted and recombinant fusion expression assays were conducted. Experiments verified that FCP promoted cell migration and adhesion and maintained cell stemness by binding integrin β3. Its effects could be blocked both by integrin β3 silencing and employing the integrin β3 inhibitor Cilengitide. Therefore, FCP may be used in the in vitro culture of stem cells, such as hematopoietic stem cells and other stem cells with high expression of integrin β3, or even in the field of tissue regeneration engineering.

## Materials and methods

### Materials

Vectors *pPICZαA* (Invitrogen, Guangzhou, China) and *P. pastoris strain X33* (Invitrogen, Guangzhou, China, Invitrogen™ C18000, ATCC® 28,485™) were used for cloning and heterologous expression. PCR purification kits, gel extraction kits, and micro preparation kits were purchased from Tiangen (Beijing, China). CC (a collagen-like protein, designed from COLA1, ID: BC036531.2, 2120-2164nt) was artificially synthesized by GL Biochem (Shanghai, China). FN10(ID: U42594.1, 633-770nt) was recombinantly expressed in our Lab (Fig. S2).

ECV304 (Human umbilical vein endothelial cells, ATCC® CRL-1998) and ECV304-eGFP (Human umbilical vein endothelial cells-enhanced green fluorescent protein, ATCC® PCS-100–010) cells were purchased from the Chinese Academy of Sciences (Shanghai, China). They were cultured in Roswell Park Memorial Institute 1640 medium (RPMI 1640, Gibco, Carlsbad, CA, USA) containing 10% fetal bovine serum, penicillin (100 I.U./mL), and streptomycin (100 μg/mL) (Sangon Biotech, Shanghai, China). Rat bone marrow mesenchymal stem cells (rBMSCs) were extracted in our laboratory approved by the Animal Care Committee of Jinan University (JNU20200826-11) and were performed in accordance with animal ethics guidelines of Agricultural Animals for Research and Teaching at Jinan University. rBMSCs were cultured in Dulbecco’s modified Eagle’s medium (DMEM, Gibco) supplemented with 10% fetal bovine serum, penicillin (100 I.U./mL), and streptomycin (100 μg/mL).

### Construction and identification of FCP

The cDNA encoding FCP was inserted into the *pPICZαΑ* vector to give a recombinant plasmid named *pPICZαΑ-FCP*; then, the *pPICZαΑ-FCP* was transformed into *P. pastoris strain X33*. Zeocin-resistant clones were selected on YPD plates containing 0.1 mg/mL of Zeocin to obtain different copies of the integrated DNA of FCP. All strains were cultivated overnight at 30 °C in 5 mL of YPD medium, and 1% of the culture was transferred to 25 mL of YPG in a 250 mL baffled flask. The cells were grown at 30 °C in a shaking incubator until the OD600 reached 1.5. The cell pellets were then induced in YPM. At the same time, methanol (0.5, 1.0, 1.5, 2.0%) was added to the culture, and 1-mL samples were collected at 0, 24, 48, and 72 h after induction. The expressions of proteins were monitored by SDS-PAGE. FCP proteins were purified using the Ni Sepharose 6 Fast Flow column combined with gel filtration Sephadex G-25. Polymerase chain reaction (F: 5′-*CATGGTGCTCCAGGTGCCCCTGGATC*-3′, R: 5′-*TGGCTTATCAATTTCAGTTCTGTAG*-3′), agarose gel electrophoresis, and western blot (His-Tag Antibody, Affinity, Biosciences, Changzhou, China), etc., were used to verify the authenticity of FCP. Expanded culture of highly expressing P. pastoris strain X33 in 2L YPD media was performed. Additionally, the circular dichroism (CD) spectrum of FCP was measured with a Chirascan plus Circular Dichroism Spectrophotometer (Applied Photophysics Ltd., Leatherhead, Surrey, UK).

### Cell migration assay: scratch wound assay

An in vitro scratch wound healing model was used to assess the cell migration induced by FCP. Briefly, ECV304 cells were plated in 12‐well plates and cultured until approximately 95% confluence before the scratch study was undertaken. A scratch was created with a 1 ml sterile pipette tip. After scratching, cells were washed using PBS to remove cell debris caused by the scratch. Another 1 mL treatment solution (250 nmol/L FCP, or FN10, or CC in 1640 medium containing 1% FBS, respectively) was added to cells. Three images of the scratch area were photographed using an inverted microscope (Olympus IX70, Tokyo, Japan) at 0, 18, and 36 h. The ImageJ software was used to determine percentage closure (%).

In another separate experiment, ECV304 cells were treated with or without FCP, integrin β3 siRNA, and NC group (negative control: cells transfected with scrambled siRNA), respectively. All operations are performed as above.

### Cell adhesion assay: crystal violet staining

Crystal violet staining was performed to assess the adhesion of FCP. Briefly, culture plates were coated with 250 nmol/L of FCP or Cilengitide overnight at 4 °C. Next, the plates were washed three times with PBS. Subsequently, ECV304 (1.0 × 10^5^ cells/mL) were seeded on tissue culture plates (96-well) and allowed to attach for 4 h at 37 °C. After incubation, non-attached cells were removed by three rinses with PBS. The remaining cells were fixed with 2% paraformaldehyde for 20 min and stained with 1% crystal violet (Solarbio, Beijing, China) for 20 min.

### Immunofluorescence staining assay

After adhesion, cell spread areas were characterized by vinculin and TRITC Phalloidin and detected by immunofluorescence staining assays via a confocal microscope. Briefly, ECV304 (2.0 × 10^5^ cells/mL) were seeded on plates (24-well) and calculated approximately 95% confluence. Then, after being treated with different test substances for 24 h, we discarded culture supernates and washed ECV304 cells using PBS 3 times, following fixed them in 4% paraformaldehyde. In the next moment, washed with PBS and then permeabilized with ice-cold 0.5% Triton X-100. The cells were blocked with PBS containing 2% bovine serum albumin, then incubated with primary antibody of vinculin (Proteintech, Wuhan, China, Cat^#^26,520–1-AP, 1:100) overnight at 4 °C; after that, Alexa Fluor 488 goat anti-rabbit IgG (Sigma, UK, Ca^#^SLBX2002, 1:80) were co-incubated at room temperature for 30 min. Next, TRITC Phalloidin (Solarbio, Beijing, China, Cat^#^CA1610, 1:200) was incubated at room temperature for 10 min. Finally, the sections were stained with 4′,6-diamidino-2- phenylindole (DAPI) (Cell Biolabs INC, San Diego, CA, Cat^#^112,002, 1:1000) and imaged with a confocal laser scanning fluorescence microscope (LSM700, Zeiss, Wetzlar, Germany).

There were three separate immunofluorescence staining assays. The test substances of the first immunofluorescence staining assays were FCP, CC, and FN10 (250 nmol/L). ECV304 cells were treated with FCP, integrin β3 siRNA, or scramble siRNA for 24 h separately in the second separated assays. In the last separate experiment, ECV304 cells were treated with FCP, Cilengitide, and FCP combined with Cilengitide (250 nmol/L) for 24 h. All test corresponding control groups were given.

### Tube formation assay

The tube formation assay was performed with ECV304-eGFP. Briefly, 96-well plates were coated with 50 μL/well matrigel, then polymerized at 37 °C for 30 min. Then, ECV304-eGFP (3 × 10^4^) were added to each well and incubated at 37 °C and 5% CO_2_ for 12 h. Cells were respectively treated with or without FCP, FN10, and CC (250 nmol/L) for 4 h. Then, tube formation was observed and captured with an inverted microscope (Olympus IX70, Tokyo, Japan).

### Protein modeling and molecular docking

The structural modeling of FCP was performed using bioinformatic webservers such as SWISS-MODEL (https://swissmodel.expasy.org/) (Waterhouse et al. 2018), I-TASSER (https://zhanggroup.org/I-TASSER/) (Yang and Zhang 2015), RoseTTAFold (https://robetta.bakerlab.org/) (Baek et al. 2021) and Alphafold2 (https: // colab.research.google.com / github / deepmind / alphafold / blob / main / notebooks / AlphaFold.ipynb) (Jumper et al. 2021). These webservers cover different modeling methods including homology modeling, threading modeling, deep learning-based ab initio modeling. FCP structure model was evaluated and verified using Ramachandran Plot (Carugo and Djinovic-Carugo 2013), Errat (Colovos and Yeates 1993), Prove (Pontius et al. 1996), Whatcheck (Hooft et al. 1996) and Verify3D (Bowie et al. 1991) evaluation procedures by SAVES6.0 (https://saves.mbi.ucla.edu/).

To explore the molecular interaction, the PatchDock server (http://bioinfo3d.cs.tau.ac.il/PatchDock/php.php) (Schneidman-Duhovny et al. 2005) was used to simulate the interaction between FCP (the highest quality model) and integrin β3 (PDB ID: IJV2).

Utilizing the FireDOCK server (http://bioinfo3d.cs.tau.ac.il/FireDock/) to screen the docking results from PatchDock. Finally, 3D and 2D plot analyses of the protein interaction surface through PyMoL v.2.5.1 and Ligplot^+^v.2.2 respectively.

### Surface plasmon resonance (SPR)

The interactions between FCP and integrin β3 were examined by SPR (Nicoya Life Science, Waterloo, Canada) (Xie et al. 2019). FCP was fixed on an NTA sensor chip by capture coupling. Integrin β3 (X–Y Biotechnology, Shanghai, China) were injected sequentially into the chamber in PBS running buffer, and an Open SPR detected the interactions of FCP with the fixed small molecules at 25 °C. The binding and disassociation times were 250 s, the flow rate was 20 μL/s, and the chip was regenerated with hydrochloric acid (pH 2.0). A one-tone diffusion-corrected model was fitted to the wavelength shifts corresponding to the various drug concentrations. The data were retrieved and analyzed with the TraceDrawer software.

### Integrin β3 siRNA transfection

Integrin β3 siRNA were transfected into ECV304 cells further to validate the interaction of integrin β3 and FCP. Integrin β3 siRNAs were synthesized by (Ribio Biotechnology Co., Ltd., Guangzhou, China) three different target sequences of the siRNA as presented in Table S1. The optimal siRNA and its concentration were determined by the efficacy of transcriptional suppression, which RT-qPCR and western blot analysis verified.

Shortly, the ECV304 cells (2 × 10^5^ cells per well) in the logarithmic growth phase were seeded in 6-well plates and cultured overnight, then transfected with integrin β3 siRNA or scramble siRNA, respectively according to the manufacturer’s protocol of Ribio. Transfected cells were incubated for another 24 h and treated with FCP; then, scratch wound assay and immunofluorescence staining assay were used to examining the effects of FCP.

### Clone formation test

rBMSCs (1 × 10^3^ cells per well) were inoculated into a 6-well plate precoated with FCP, Cilengitide, and FCP combined with Cilengitide (250 nmol/L), respectively; the group that did not precoat with anything as the blank control. The cells were cultured continuing for 14 days. After that, the cells were washed three times with PBS and incubated with 1 mL of crystal violet staining solution (Solarbio, Beijing, China) for 30 min. After being washed three times, the cells were observed under an optical microscope (Nikon, Japan) and photographed. At the same time, cells on another independent 6-well plates were collected to detect stemness-related genes: NANOG; REX1; PPAR-γ2; ALP; SOX9, and integrin β3.

### Reverse transcription-quantitative PCR (RT-qPCR)

Total RNA from rBMSCs subjected to different treatments was extracted using TRIZOL reagent (Tiangen Biotechnology, Beijing, China). To obtain cDNA, 1 μg of RNA was reverse transcribed by using the reverse transcription kit (Tiangen Biotech, Beijing, China). An equal volume of cDNA was then used for RT-qPCR using the SYBR-Green Quantitative PCR kit (Bio-Rad, Hercules, CA, USA) and the CFX96 Touch Real-Time PCR Detection System (Bio-Rad, Hercules, CA, USA). The rat-specific primers were as follows: NANOG; REX1; PPAR-γ2; ALP; SOX9; and integrin β3. All primers were purchased from BGI (Shenzhen, Guangdong, China). Relative expression levels of genes were examined by 2^−ΔΔCt^ method, with GAPDH taken as the normalization. The sequences of primers were provided in (Table S3).

### Statistical analysis

All data are expressed as mean ± standard deviation (*SD*) of at least three independent experiments. Statistical analyses were performed using the GraphPad Prism 6 software (GraphPad Software Inc, La Jolla, CA, United States). Differences between more than two groups were analyzed using one-way ANOVA followed by Tukey s HSD comparison test. Statistical significance was set at *P* < 0.05.

## Results

### Construction, expression, and purification of recombinant protein FCP

**Fig. 1:**
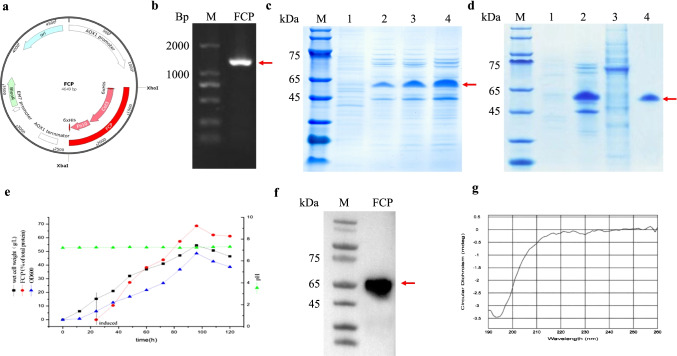
Construction and identification of FCP. **a** Construction schematic of the recombinant *pPICZαΑ-FCP-his* plasmid. **b** Nucleic acid electrophoresis of a recombinant *pPICZαΑ-FCP-his* plasmid. M: DNA Ladder 2000. **c** FCP was induced by methanol for 0 h, 24 h, 48 h, and 72 h at 30℃. M: middle molecular weight protein markers; lane 1 supernatant of FCP before induction; lane 2, 3, and 4 supernatants of FCP after induction for 24 h, 48 h, and 72 h. **d** Purification of FCP; lane 1: before induction; 2: after FCP induction; 3: flow through peak; 4: FCP elution. **e** In expanding the culture of recombinant protein FCP with a 2L scale. **f** Western blotting analysis of recombinant FCP. M: molecular weight markers. **g** CD spectra of FCP solutions

The coding sequence of the FCP fusion, FCP, was inserted into the *pPICZαΑ* plasmid distal to the methanol-inducible AOX1 promoter, which can be repressed by glycerol and activated by methanol. The resulting plasmid, named *pPICZαΑ-FCP-his*, contained a zeocin resistance gene that was used for positive clone screening. Nucleic acid electrophoresis of the recombinant plasmid is shown in Fig. 1a and b. The positive *P. pastoris strain X33* was treated with methanol for 0 h, 24 h, 48 h, and 72 h at 30 °C. FCP expression was the highest in the supernatant (verified with WB, Fig. 1f) following 72 h of methanol induction, reaching approximately 0.6 mg/ml (Fig. 1c). SDS–PAGE was used to analyze the purification efficiency of FCP (Fig. 1d). During culture expansion of the recombinant protein FCP to a 2 L scale, the OD value, bacterial liquid pH, bacterial wet weight, and FCP expression were detected every 12 h. According to the biological characteristics, a growth curve was drawn and applied to optimize FCP fermentation conditions (Fig. 1e). Characterization of the secondary structure of FCP in solution was carried out by CD spectroscopy, which utilizes the differential absorption of left- and right-handed circularly polarized light in an asymmetric environment to assess secondary structure (Correcirc and Ramos 2009). CD spectroscopy results are shown in Fig. 1g. FCP displays a characteristic CD spectrum with a negative peak at approximately 198 nm and a small, broad positive peak at approximately 220 nm, which corresponds to a random coil structure (Correcirc and Ramos 2009; Wei et al.^ 2014^). These results indicate that the FCP structure is predominately a random coil, consistent with our predictive model.

### FCP promotes ECV304 cell adhesion, wound healing, and vascularization better than FN10 and CC

**Fig. 2:**
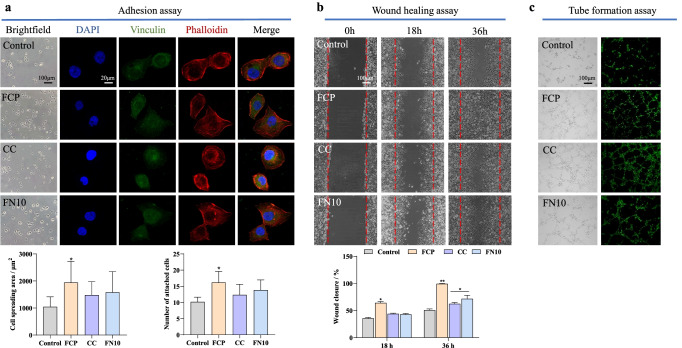
Cell biological activity of FCP, CC, or FN10. **a** ECV304 cells were treated with FCP, CC, or FN10 for 24 h. Focal adhesion was detected by immunofluorescence staining for vinculin (green), phalloidin (red), and DAPI (blue). Bright-field micrographs. Scale bar = 100 μm, 20 μm. The graph shows the number of adherent ECV304 cells and the quantitative calculation of cell spread area. **b** Images were obtained at 0, 18, and 36 h after wound creation in vitro migration assay on FCP, CC, or FN10. Scale bar = 100 μm. The graph shows the quantitative analysis of wound closure of ECV304 cells cultured on FCP, CC, or FN10 for 18 and 36 h. **c** Tube formation by ECV304-eGFP cells. Scale bar = 100 μm. *n* = 3, mean ± *SD*, *P < 0.05, **P < 0.01 vs. control

To investigate the effect of FCP, CC, or FN10 on cell adhesion, an immunofluorescence assay was performed using vinculin, phalloidin, and DAPI staining (Fig. 2a). The results of the adhesion assay and immunofluorescence staining showed that the cell spreading area of ECV304 cells treated with FCP (250 nmol/L) was significantly increased compared with that of the control groups (Fig. 2a, *P* < 0.05). A similar trend was observed in the number of attached cells (Fig. 2a, *P* < 0.05). After 36 h of FCP treatment, the wound was almost completely closed in the wound healing assay, while others were still open (Fig. 2b). After a 36-h incubation, a significantly higher percentage (*P* < 0.01) of wound closure was observed in the FCP groups (99.32% ± 0.69%) than in the control groups (50.68% ± 2.31%), (Fig. 2b). Similarly, in the tubule formation assay, FCP and FN10 also exhibited the best ability to vascularize (Fig. 2c).

### FCP binds integrin β3

**Fig. 3:**
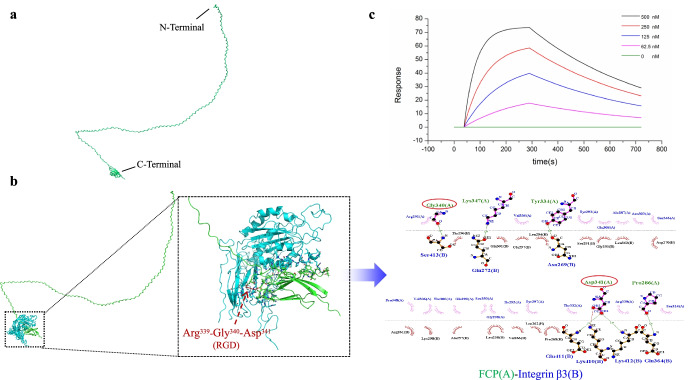
FCP model construction and molecular docking with SPR validation. **a** Structural modeling of FCP using Robetta server. The collagen fragments at the N-terminus of FCP are mainly random coils, while the FN fragments at the C-terminus are mainly β-sheets. **b** The 3D and 2D plot of the molecular interaction of FCP-Integrin β3 complex. The FN fragment of FCP forms a stable combination with Integrin β3, and the key interacting residues of FCP include Pro286, Try334, Gly340, Asp341, Lys347, which marked by red circles belong to the RGD sequence. **c** Interaction detection between different concentrations of FCP and integrin β3 passes SPR

Different prediction models of FCP were validated using Ramachandran Plot, Whatcheck, and Verify3D by the SAVES 6.0 online server. Validation statistics showed that RoseTTAFold best predicted the structure of FCP compared with the other methods, evidenced by the presence of the maximum residues (83.6%) in the most favored regions of the Ramachandran plot, 100% of the residues with an average 3D-ID score ≥ 0.2, and the largest proportion of green regions (68.1%) in Whatcheck (Fig. 3a, Table S2). The best predicted model (Fig. 3b) showed that FCP has an extensive range of random coil and partial β-sheet structures (Fig. S3), consistent with the results of CD (Fig. 1g), in which there were 48% β-sheets and 41% random coils in FCP.

Based on these results, the RoseTTAFold model was used for molecular docking. After protein–protein docking via PatchDock and filtering the docking results by FireDock, the stable docking complex of FCP-integrin β3, with the highest binding energy of − 15.68 kcal/mol, was obtained. The 3D plot of the docking complex showed that the C-terminal FN10 region of FCP formed a relatively stable complex with integrin β3 (Fig. 3b). More specifically, the 2D plot of the interaction surface visually showed that a total of 5 amino acid residues of FCP (Pro286, Try334, Gly340, Asp341, Lys347) and 7 amino acid residues of integrin β3 (Asn269, Gln272, Glu364, Lys410, Glu411, Lys412, Ser413) served as key sites involved in protein–protein binding (Fig. 3b). It is worth mentioning that Gly340 and Asp341 of FCP belong to the RGD sequence.

SPR was then executed to validate the computer performance prediction. The results showed that FCP interacted with integrin β3 in a dose-dependent manner. The SPR assay showed an affinity constant of 5.37 × 10^–8^ M (Fig. 3c), indicating a strong binding affinity between FCP and integrin β3.

### Integrin β3 knockdown-reduced FCP-induced ECV304 cell migration and adhesion

**Fig. 4:**
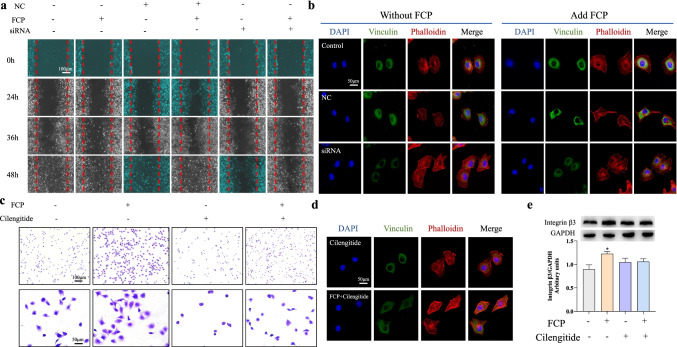
Integrin β3 knockdown-reduced migration and adhesion of ECV304 induced by FCP. **a** Images obtained at 0, 24, 36, and 48 h after wound creation in vitro migration assay on NC, FCP, or siRNA. **b** ECV304 cells were treated with or without FCP, negative control (negative control: cells transfected with scrambled siRNA), or siRNA. Focal adhesion was detected by immunofluorescence staining for vinculin (green), phalloidin (red), and DAPI (blue). Scale bar = 50 μm. **c** Optical micrographs of crystal violet–stained ECV304 cells adhering to monolayers. Scale bar = 100 μm, 50 μm. **d** Immunofluorescence staining assay of ECV304 cells was treated with FCP or Cilengitide. **e** The levels of ECV304 cells integrin β3 expression were determined by western blot analysis. *n* = 3, mean ± *SD*, *P < 0.05 vs. control

Integrin β3 expression was knocked down with 50 nM integrin β3 siRNA treatment in ECV304 cells and confirmed by RT–qPCR (Fig. S1a, b) and western blot (Fig. S1c, d). Following integrin β3 knockdown, wound healing slowed, and the expression of both Vinculin and Phalloidin decreased (Fig. 4a, b). Interestingly, this phenomenon was reversed after adding FCP (Fig. 4b).

### Cilengitide reduced endothelial migration and adhesion induced by integrin β3

Cilengitide, an RGD pentapeptide inhibitor of integrin αvβ3, blocks integrin ανβ3-mediated endothelial cell attachment and migration (Hariharan et al. 2007). In the crystal violet assay, cell adhesion was significantly promoted by FCP compared with control and Cilengitide-treated groups (Fig. 4c). More cells attached in the FCP/Cilengitide groups than in the Cilengitide groups (Fig. 4c). Immunofluorescence showed weaker Vinculin and Phalloidin fluorescence following Cilengitide treatment. However, upon simultaneous treatment with FCP and Cilengitide, Vinculin and Phalloidin fluorescence were enhanced (Fig. 4d). Western blot showed that Cilengitide treatment of ECV304 did not affect integrin β3 expression (Fig. 4e), so we speculate that FCP cannot play the role of promoting cell adhesion due to competitive with Cilengitide to bind integrin β3. FCP could reverse the inhibitory effect of Cilengitide.

### rBMSC enumeration and colony characterization using a standard CFU-F assay

**Fig. 5:**
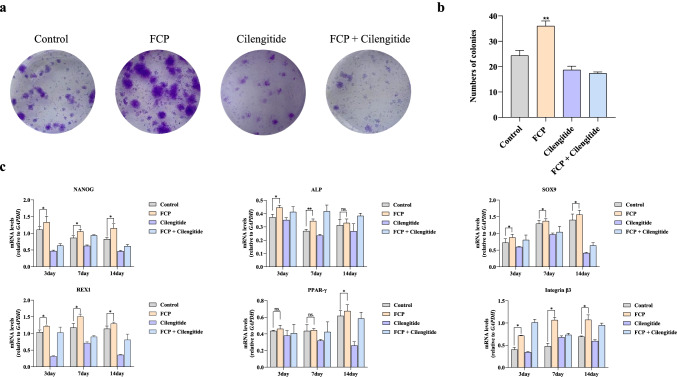
Experimental study of cell stemness of rBMSCs and trilineage differentiation-related genes after treatment with FCP or Cilengitide. **a** Clone formation test of rBMSCs. **b** Quantitative analysis of the numbers of colonies. **c** RT-qPCR analysis of NANOG, REX1, PPAR-γ2, ALP, and SOX9 and integrin β3 expression in rBMSCs were treated with FCP or Cilengitide for 3, 7, 14 days, *n* = 3, mean ± *SD*, *P < 0.05, **P < 0.01, ns, means no significant differences, *P* > 0.05 vs. control

The clonality and proliferation ability of rBMSCs are reflected by the CFU-F assay. rBMSCs of all groups formed cell colonies. Colony formation of each group was counted under a light microscope after crystal violet staining. The number and clone formation of rBMSCs treated with FCP were significantly higher than that of the control (Fig. 5a, b). Interestingly, when Cilengitide inhibited integrin β3, the colony formation ability of rBMSCs decreased significantly (Fig. 5a, b). Western blot showed that integrin β3 was highly expressed in rBMSCs (Fig. S4), and Cilengitide did not affect this expression (Fig. 4e). Collectively, these data suggest that FCP activate integrin β3 on the surface of rBMSCs and may act via integrin β3 binding and activation to improve rBMSCs’ colony-forming ability.

FCP administration increased expression of the stemness-related genes NANOG and REX1 compared to control, and these higher gene expression levels were maintained within 14 days following FCP administration (*P* > 0.05). Gene expression levels of both SOX and ALP, gene markers of chondrogenic and osteogenic differentiation abilities of rBMSCs, were higher than others, especially compared to gene expression levels in the control group (*P* < 0.05). However, gene expression of PPAR-γ2, an adipogenic gene, was similar in FCP-treated and control groups (*P* > 0.05). When Cilengitide was used to inhibit integrin β3, the expression of all genes was significantly downregulated. After treatment with both FCP and Cilengitide simultaneously, the related genes were upregulated compared with those in the Cilengitide groups (Fig. 5c).

## Discussion

The key to developing biomaterials is to design biomaterials that contain specific domains which can interact with biological systems to meet various medical and health needs. Protein-functional materials have a distinct advantage in medical and green manufacturing due to their good biocompatibility, biodegradability, multifunctionality, and environmental friendliness.

This study designed a novel recombinant human fusion protein, FCP, with specific protein domains that bind the integrin β3 receptor to communicate with specific stem cells. FCP is composed of FN10 and CC. FN10 is a functional domain of FN that contains the RGD sequence. CC is a recombinant collagen-like protein, developed by our laboratory, that promotes the migration and adhesion of NIH/3T3 cells (Cheng et al. 2020). In a full-thickness animal wound model, CC dressing significantly promotes wound healing and angiogenesis. However, CC exhibits thermal instability, a characteristic that leaves great room for improvement. Thus, in the present study, the CC fragment was combined with FN10 to construct and express FCP protein, improving the performance of the recombinant protein to meet the needs of tissue engineering. Compared with the individual CC and FN10 components, FCP promotes improved cellular proliferation, adhesion, and angiogenesis. The comparative analysis of three proteins shows a satisfying result. All aspects of the performance of FCP improved significantly.

To better understand the structure and function of FCP, the FCP protein structure was predicted using various protein modeling methods. Model quality assessment showed that the FCP model based on ab initio prediction without a homologous template, which was optimized by deep learning, reflected the FCP structure most realistically. Various modeling approaches were tested in optimally constructing a stable FCP structure. The homology and threading methods accurately and efficiently construct the spatial conformations of target proteins with homologous sequences and similar topologies based on the vast amount of known protein structure information obtained from the database. However, these methods are not applicable to unknown proteins that lack template information (Waterhouse et al. 2018; Yang and Zhang 2015). In contrast, for newly designed proteins, such as FCP, a template-independent ab initio prediction modeling approach is best for constructing stable protein models. However, this modeling method is computationally resource intensive and time-consuming. The Alphafold2 server based on a deep learning algorithm was also tested for protein structure modeling; however, the model quality was not satisfactory (Supplement Fig. 3). We speculate that this may be due to the largely irregular curl structure of FCP leading to poor construction.

After obtaining a high-quality structural model of FCP, molecular docking studies of FCP and integrin β3 were performed. Computer simulations showed that the FN10 C-terminus within the FCP protein could form a stable complex with the extracellular segment of integrin β3, consistent with our original design goal. According to these results, modification of the residues on the docking interaction surface of the FCP protein and integrin β3 may potentially enhance the affinity of FCP for integrin β3.

The high affinity of FCP for integrin β3 was verified by SPR assay. Integrin β3 protein, captured on an NTA chip, bound FCP with an affinity constant of 5.37 × 10^–8^ M, as determined by the SPR assay. Thus, the integrin β3 protein strongly binds the FCP protein (10^–8^ ~ 10^–10^ M). The SPR technique is a powerful tool in the study of target molecule interaction and has the advantages of low cost and direct and quantifiable results. However, because SPR is sensitive to interfering factors within the sample, it represents an indirect interaction between proteins.

Collectively, these data suggest that integrin β3, an important receptor on the surface of ECV304 cells, may be a key target for FCP to exert its biological effects. By knocking down integrin β3 expression in ECV304 cells and competitively blocking integrin β3 binding sites with Cilengitide, FCP-induced angiogenesis, migration, and adhesion of ECV304 cells were reduced. rBMSCs were chosen as the model system in the present studies due to ethical constraints and other difficulties in obtaining hHSCs. Considering that rBMSCs are one of the most widely used stem cells in the field of tissue engineering (Chen et al. 2018; Li et al. 2019) and that integrin β3 is highly expressed on the surface of the BMSC cytomembrane (Pei et al. 2011), rBMSCs were utilized in the cloning formation assays. FCP enhanced the colony-forming ability of rBMSCs, and this effect was significantly decreased by Cilengitide pretreatment. After treatment with both FCP and Cilengitide, the related genes were upregulated compared with those in the Cilengitide only group. Likewise, the stemness-related genes NANOG and REX1 expression was increased following FCP administration when compared to control, and this increase remained 14 days after treatment. The gene expression levels of both SOX and ALP, markers of chondrogenic and osteogenic differentiation abilities of rBMSCs, were both higher than that observed in other genes. However, the gene expression of PPAR-γ2, an adipogenic gene, was similar to that of the control (*P* > 0.05). This finding is difficult to explain, but we speculate that it is related to the function of integrin β3. Further study of these results was outside the scope of the current study due to laboratory constraints. For a sample, we are unable to test karyotypic stability after transfer generation.

Functional recombinant proteins that are used as replacements for native proteins have many unique characteristics. Their high molecular weight, high-frequency amino acids, and special posttranslational modifications serve as bottlenecks in the artificial cell synthesis field and lead to low expression rates and poor adaptation between functional elements and host cells. Functional recombinant protein structures may be unstable or ineffective, limiting efficient production and application of protein-functional materials. Therefore, it is particularly important to ab initio design protein molecules and construct a cell factory to realize functional directional enhancement and industrial production of these functional recombinant proteins. Fibronectin and collagen are principal structural proteins of the ECM. By combining their ECM functions allows us to more comprehensively understand the molecular structure of ECM and its bioactivity in order to design new therapeutic compounds in the future. FCP combines the FN10 (one of fibronectin functional domain-containing RGD sequence) and CC (a kind of human-like collagen) proteins, which have an ideal domain to provide mechanical support for cells. The FCP bionic ECM may provide a microenvironment to support stem cell localization, proliferation, differentiation, and stemness maintenance. In the future, FCP may be used in the in vitro culture of stem cells, such as hematopoietic stem cells and other stem cells with high expression of integrin β3, or even in the field of tissue regeneration engineering.

## Supplementary Information

Below is the link to the electronic supplementary material.

Supplementary file1 (PDF 564 KB)

## Data Availability

The raw data supporting the conclusion of this article will be made available by the authors, without undue reservation.
