# Supplementary material for: Recombinant expression a novel fibronectin—collage fusion peptide modulating stem cell stemness via integrin β3
Source: Appl Microbiol Biotechnol. 2022 May 20;106(9-10):3765–76. doi: 10.1007/s00253-022-11965-4 (PMC9151557; doi:10.1007/s00253-022-11965-4)
Supplement: Supplementary file 1 — Supplementary file1 (PDF 564 KB) [file 253_2022_11965_MOESM1_ESM.pdf]

Applied Microbiology and Biotechnology

Recombinant expression a novel fibronectin - collage fusion peptide modulating stem cell stemness via integrin β3

Xin Luo[1†], Dezhi Geng[1†], Qirong Zhang[1], TaoYe[2], Yifan Zhang[2], Ziyi Li[2], Yadong Huang[1,2] and Qi Xiang[1,2*]

[1]Institute of Biomedicine and Guangdong Provincial Key Laboratory of Bioengineering Medicine, Jinan University, Guangzhou, China,

[2]Biopharmaceutical R&D Center of Jinan University, Guangzhou, China

Corresponding author: Institute of Biomedicine and Guangdong Provincial Key Laboratory of Bioengineering Medicine, Jinan University, Guangzhou 510632, China. Tel.: +86-208-556-3234.

E-mail address: txiangqi@jnu.edu.cn (Qi. Xiang).

[†]These authors have contributed equally to this work

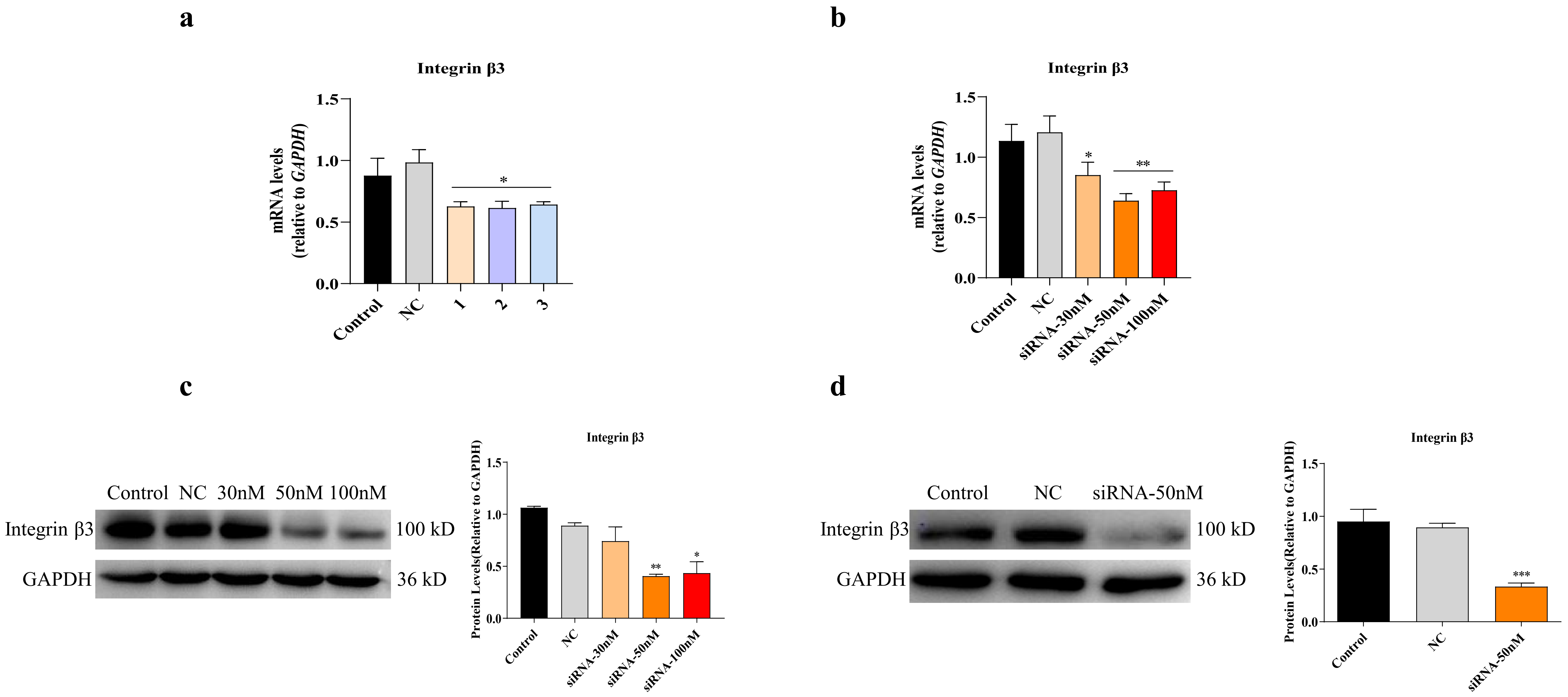

Fig. S1 Screening the optimal fragment and concentration of siRNA. **a** The mRNA level of integrin β3 in ECV304 cells transfected with 50 μm different fragment (Table S1) compared with the NC group (negative control: cells transfected with scrambled siRNA). **b** The mRNA level of integrin β3 in ECV304 cells transfected with different concentrations of fragment 2. **c** The level of integrin β3 in ECV304 cells transfected with different concentrations of siRNA was examined by Western blot analysis. GAPDH was used as a control. **d** The level of integrin β3 in ECV304 cells transfected with 50nM siRNA (fragment 2) for 24 hours by Western blot analysis. n = 3, mean ± SD, $^{*}P < 0.05$, $^{**}P < 0.01$, $^{***}P < 0.001$ *vs.* Control group.

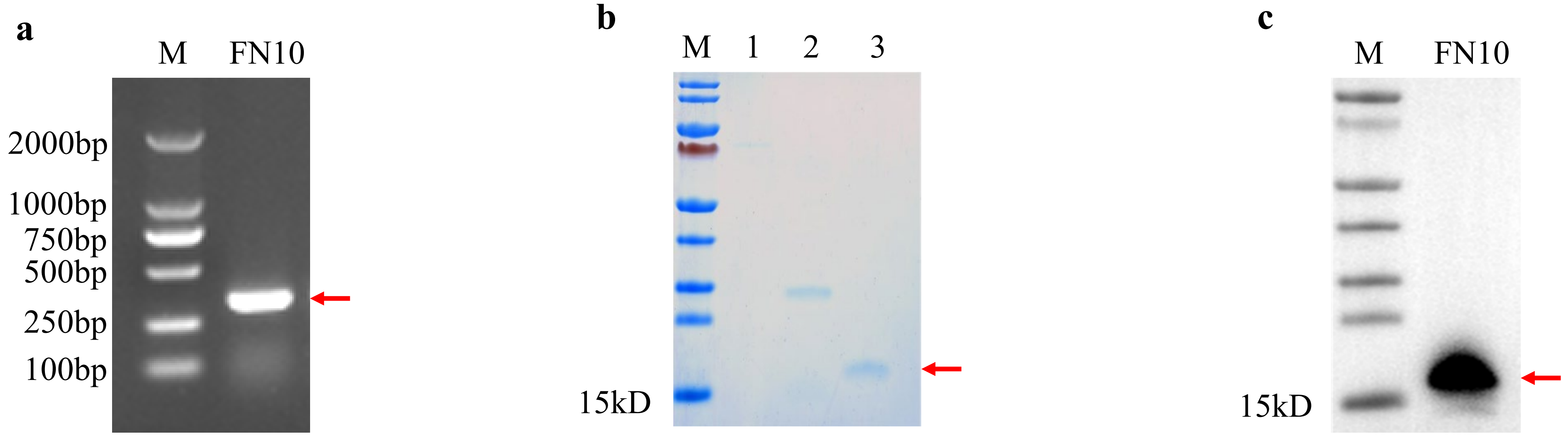

Fig. S2 The identification and purification of FN10. **a** Nucleic acid electrophoresis of FN10 plasmid; M: DNA Ladder 2000. **b** Purification of FN10; lane 1: flow-through peak; lane 2: wash to remove impurities; lane 3: FN10 elution. **c** Western blotting analysis of recombinant FN10; M: Molecular weight markers; arrow points to FN10.

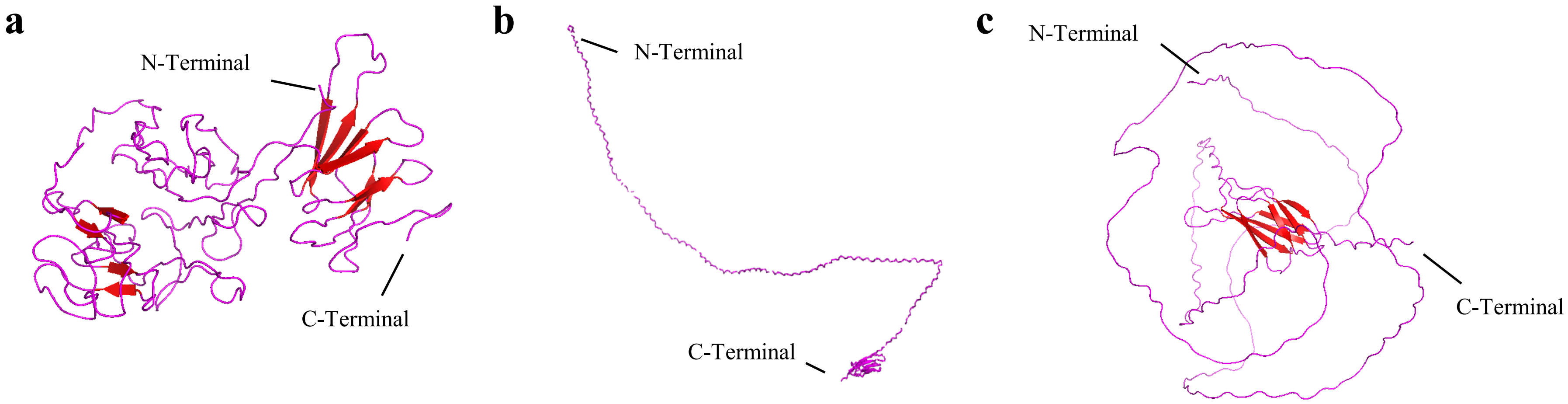

Fig. S3 Structural modelling of FCP using multiple servers. **a** the I-TASSER model for FCP. **b** the Robetta model of FCP. **c** the Alphafold2 model of FCP.

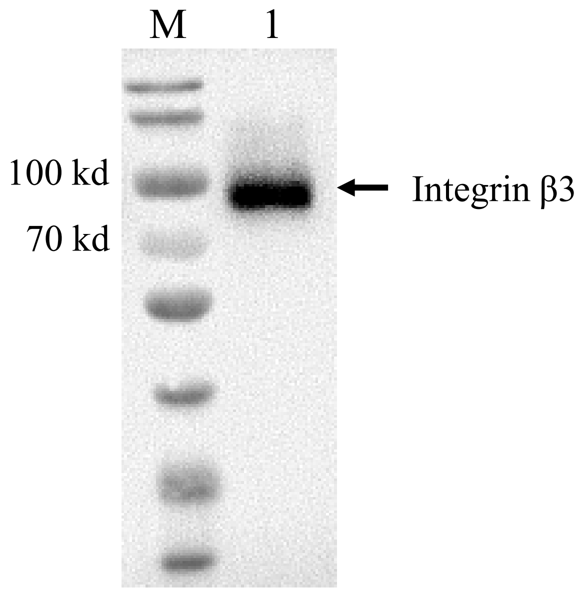

Fig. S4 Western blot analysis showed Integrin β3 expression in rBMSCs. M: middle molecular weight protein markers; lane 1 The level of integrin β3 in rBMSCs.

Table S1 Target sequences of the siRNA.

siRNA	Target sequences
**h-Integrin β3-1**	5′- GATGCAGTGAATTGTACCT -3′
**h-Integrin β3-2**	5′- ACCGGCTACTACTGCAACT -3′
**h-Integrin β3-3**	5′- GAGCCCTACATGACGAAAA -3′

Table S2 Ramachandran plot statistics, Verify3D and the percentage of Whatcheck green regions for FCP models obtained by different servers.

	Ramachandran plot				Verify 3D score	Whatcheck green regions(%)
	MF regions(%)	AA regions(%)	GA regions(%)	DA regions(%)		
I-TASSER	52.7% core	38.6% allow	6.3% gener	2.4% disall	90.56	53.2%
Robetta	83.6% core	9.7% allow	2.4% gener	4.3% disall	100.00	68.1%
Alphafold2	35.2% core	16.3% allow	24.7% gener	23.8% disall	92.41	40.4%

Table S3 Rat-specific primer sequences used for quantitative real-time PCR

Gene	Primer
**NANOG-F**	5′- CTGCCTCTCCTCCGCCTTCC-3′
**NANOG-R**	5′-CTCGTCAGCCTCGGGACCAG-3′
**Rex1-F**	5′- CACTGTCCTCCTCCTCGTCCTC-3′
**Rex1-R**	5′-CGTTGAAGATCCGCAGGCACTC-3′
**ALP-F**	5′-CACGGCGTCCATGAGCAGAAC-3′
**ALP-R**	5′-CAGGCACAGTGGTCAAGGTTGG-3′
**PPAR-γ2-F**	5′- CAGCACTGAGGAAAGCTGGT-3′
**PPAR-γ2-R**	5′- GTGCTCTGTGACAATCTGCCTGAG-3′
**Sox9-F**	5′-GCAAAGCTGCGGTGCTATG -3′
**Sox9-R**	5′-TCACACAAGTCACCCCTTCTC-3′
**Intergrin β3-F**	5′-TCATCTGGAAACTCCTCATCAC-3′
**Intergrin β3-R**	5′-GTAGACGTGGCCTCTTTATACA-3′
**GAPDH-F**	5′-GAGCGCAAGTACTCTGTGTG-3′
**GAPDH-R**	5′-AACGCAGCTCAGTAACAGTC-3′
